# Topiramate modulates trigeminal pain processing in thalamo-cortical networks in humans after single dose administration

**DOI:** 10.1371/journal.pone.0184406

**Published:** 2017-10-09

**Authors:** Julia M. Hebestreit, Arne May

**Affiliations:** Department of Systems Neuroscience, Center for Experimental Medicine, University Medical Center Hamburg-Eppendorf, Hamburg, Germany; Taipei Veterans General Hospital, TAIWAN

## Abstract

Migraine is the sixth most common cause of disability in the world. Preventive migraine treatment is used to reduce frequency, severity and duration of attacks and therefore lightens the burden on the patients’ quality of life and reduces disability. Topiramate is one of the preventive migraine treatments of proven efficacy. The mechanism of action underlying the preventive effect of topiramate in migraine remains largely unknown. Using functional magnetic resonance imaging (fMRI) we examined the central effects of a single dose of topiramate (100mg) on trigeminal pain in humans, compared to placebo (mannitol). In this prospective, within subject, randomized, placebo-controlled and double-blind study, 23 healthy participants received a standardized nociceptive trigeminal stimulation and control stimuli whilst being in the scanner. No differences in the subjective intensity ratings of the painful stimuli were observed between topiramate and placebo sessions. In contrast, topiramate significantly decreased the activity in the thalamus and other pain processing areas. Additionally, topiramate increased functional coupling between the thalamus and several brain regions such as the bilateral precuneus, posterior cingulate cortex and secondary somatosensory cortex. These data suggest that topiramate exhibits modulating effects on nociceptive processing in thalamo-cortical networks during trigeminal pain and that the preventive effect of topiramate on frequent migraine is probably mediated by an effect on thalamo-cortical networks.

## Introduction

Migraine is among the most prevalent neurological disorders [1] and certainly one of the most disabling [2]. Its severe socioeconomic impact and the harmful influence on a patient’s quality of life are alarming [3], which stresses the importance of the improvement of current treatment options. The pharmacological treatment options are either acute (abortive) or preventive (prophylactic). Preventive migraine treatment aims at reducing frequency, severity and duration of attacks. Anticonvulsants are commonly used as preventive migraine treatment with topiramate being among the most widely used. Due to the extensive body of evidence that proves its efficacy, topiramate is considered an evidence-based medication in the preventive treatment of migraine [4]. Although effective in reducing migraine attack frequency [5,6], little is known about the underlying central mode of action in migraine treatment [7]. Most of our understanding of modes and sites of action behind its therapeutic efficacy in migraine derives from preclinical animal studies, utilizing different models of trigeminal activation. Topiramate affects glutamatergic and GABAergic transmission, as well as sodium and calcium channels [8,9]. All of these mechanisms influence trigeminovascular activity and may play a role in migraine although the exact mechanism is not known [7].

Storer and Goadsby [10] showed that systemically administered topiramate partly inhibited stimulus-evoked cell firing in the trigeminocervical complex, but failed to show an inhibition after local application. The authors concluded that topiramate is likely to act outside of the trigeminocervical complex in cats. However, a study investigating the role of GABA receptors in thalamic neurons in response to nociceptive stimuli in rats finds an effect of anticonvulsants on the thalamus: Neuronal thalamic firing in response to L-glutamate injection and electrical stimulation was inhibited by GABA and valproate [11]. Using electrophysiological techniques Andreou et al. showed that topiramate inhibits nociceptive neurotransmission in the trigeminothalamic pathway of rats [12]. Activation in the ventroposteromedial (VPM) nucleus of the thalamus after electrophysiological stimulation was significantly inhibited after intravenous administration as well as after local application of topiramate into the thalamus [12]. The results indicate that neurons within the thalamus may modulate trigeminal nociceptive transmission. These data imply that topiramate exerts some of its therapeutic effect by an inhibiting effect on the thalamus.

The aim of this study was to investigate the effect of topiramate on the neural activity (measured by blood oxygen level-dependent [BOLD] responses) following a standardized experimental trigeminal pain paradigm [13,14]. Building upon the above mentioned results of animal studies we expected topiramate to have an inhibiting effect on the thalamus.

## Materials and methods

### Participants

27 healthy volunteers participated in the experiment. They were recruited using public internet advertisements in 2016. 2 participants were excluded from the analysis due to technical problems, 2 participants withdrew consent after the first session. The final sample included 23 participants (10 male; age: 19 to 34 years, M = 24.4). None of the volunteers reported a history of neurological, psychiatric or pain disorders, specifically no headache disorders. None of the subjects reported contraindications for topiramate. The study was approved by the local ethics committee (Ärztekammer Hamburg; PV4084) and participants gave written informed consent. Volunteers were remunerated for participation.

### Experimental design and task

This study had a placebo-controlled, crossover, randomized and double-blind design. The two identical sessions were separated by at least a two weeks wash-out period. Initially participants took an oral dose of either 100mg topiramate or placebo, which was followed by a waiting phase of 1 hour to reach tmax of topiramate [9]. Prior to the experimental phase, blood samples were drawn and in their first session participants completed a training session to familiarize with the task. In the experimental phase participants performed a standardized trigeminal pain paradigm in the MR scanner, that has been described in detail before [14]. In short, 4 different stimuli where applied 15 times each in a pseudorandomized order. Participants perceived either a trigemino-nociceptive stimulus (gaseous ammonia), an olfactorial stimulus (rose odor), a neutral stimulus (air puffs) or a visual stimulus (flickering checkerboard), of which all gaseous stimuli were administered through a Teflon tube to the left nostril. Following each trial, participants had to rate the stimulus intensity (0 = no pain/no sensation to 100 = highest pain imaginable / highest intensity imaginable) and pleasantness (analogous to the intensity ratings from -50 = very pleasant to 50 = very unpleasant), respectively, on a visual rating scale in the scanner.

### Medication

Participants were randomly assigned to receive an oral topiramate dose (100mg; Topamax ®; Janssen-Cilag, Neuss, Germany) or placebo in the first session, followed by at least a 2-week washout period before starting the second session with the alternate substance. Placebo pills contained medically non-effective mannitol [15,16] (Mannitol 99.5 T, highly-dispersed silicon dioxide 0.5 T) and matched the topiramate pills in appearance. Blood samples were drawn 60 min after administration to determine the topiramate plasma concentration at its peak, shortly before the start of the MR measurement. Vital signs (blood pressure, heart rate) were monitored. Possible side effects were assessed via interview.

### MRI data acquisition

Functional and structural images were acquired on a 3T Siemens Trio scanner (Siemens AG, Erlangen, Germany) using a 32-channel head coil. High-resolution structural T1-weighted images were acquired using a magnetization-prepared rapid gradient echo sequence (1 x 1 x 1 voxel size). Functional images were acquired using an echo planar imaging sequence (repetition time 2.62 seconds, echo time 30 milliseconds, flip angle 80°, field of view 220 x 220 mm). One functional imaging session consisted of ~ 900 to 1100 volumes; while each volume consisted of 40 axial slices (2 mm slice thickness, 1 mm gap). Total scantime was about 55 minutes per session for the whole experiment.

### Determination of plasma concentration

A 4.9 ml blood sample was drawn from the forearm into a vacuum tube with ethylenediaminetetraacetic acid (EDTA). Plasma was obtained by centrifugation at 1.6 rcf for 7 min at 20°C and stored at -20°C until analyzed using liquid chromatography/mass spectrometry (LC-MS/MS) [17]. Calculation of the concentration was based on an internal standard. Analysis of plasma was conducted by AescuLabor Hamburg GmbH.

### Behavioral data analysis

Behavioral responses were assessed by a visual analogue rating scale (VAS) and averaged across items per session. Data analysis was performed using SPSS Statistics version 22.00 (IBM Corp., Armonk, NY). Significant differences in the average pain intensity ratings between medication and placebo session were tested with a paired t-test. Statistical threshold was set to *p*<0.05.

### MRI statistical analysis

Data analysis was performed using statistical mapping software SPM12 (Wellcome Trust Centre of Neuroimaging, http://www.fil.ion.ucl.ac.uk/spm) using standard algorithms and parameters unless specified differently. The first 5 pictures of each session were deleted. Preprocessing included slice time correction, data realignment to the mean volume, spatial normalization into MNI (Montreal Neurological Institute) stereotactical space and spatial smoothing (8mm Gaussian kernel).

Single subject analysis was performed based on the general linear model (GLM) as implemented in SPM12. For each session five separate, experimental regressors were included in the design matrix: Four experimental conditions (ammonia, rose odor, air puffs, and visual checker board) and the button presses. These regressors were constructed by convolving stick functions at stimulus onsets with a canonical hemodynamic response function. Additional nuisance was attenuated by including six movement regressors (3 translational and 3 rotational), obtained from the realignment step, for each session into the model. The first-level design subsequently included 22 regressors, 11 for each session (placebo and topiramate). Contrast images of the comparison ammonia > air puffs were computed for both sessions (medication and placebo). Also contrast images were computed which compared nociceptive processing (ammonia > air) between medication and placebo session on individual level. Olfactorial and visual stimuli were not used for further analyses on group level.

These contrast images of each participant were then used for group statistics. Since a within-subject comparison between medication and placebo session had already been calculated on subject level, a one–sample t-test across all subjects was computed. Based on results from animal studies we were specifically interested in the effect of topiramate on the thalamus. Successively a small volume correction (SVC: p_(FWE)_ < 0.05) of the thalamus was performed using a mask obtained from the Harvard-Oxford cortical/subcortical structural atlas (http://www.cma.mgh.harvard.edu/fsl_atlas.html). Additionally the whole brain analysis of the differential contrasts between topiramate and placebo is reported at a threshold of p_(FWE)_ < 0.05 at cluster level. For clustering, the uncorrected p-threshold at voxel level of p 0.001 was used for this and the following analysis. A psychophysiological interaction analysis (PPI) [18] was calculated to determine changes in pain-related functional connectivity of the thalamus (identified as a region of interest in preceding analysis) caused by topiramate. A thalamus mask of the right and left thalamus cortex of the Harvard-Oxford cortical/subcortical atlas was used as the seed region. The GLM for the PPI included three regressors (1. the time course extracted from the seed region, 2. pain (ammonia > air) as the psychological/stimulus variable, 3. the interaction term [i.e. time series × stimulus variable]) and additionally 6 movement regressors. A one–sample t-test for the difference between sessions was computed, analogous to the above mentioned second level analysis. A threshold of p_(FWE)_ < 0.05 (at cluster level) was regarded significant.

## Results

### Behavioral data

Mean pain intensity ratings measured by the VAS scale (±SEM) where 58.3 (± 3.6) for the topiramate session and 59.3 (±4.2) for the placebo session. There was no significant difference between mean pain intensity ratings in both sessions.

### Physiological data

The mean plasma concentration of topiramate was 1.38 mg/L (SD = 0.8). Treatment-emergent adverse events were reported by 16 participants. These included mild to moderate dizziness, difficulty with concentration, paresthesia and fatigue.

### Imaging data

#### Main effects of nociceptive stimulation

**Fig 1:**
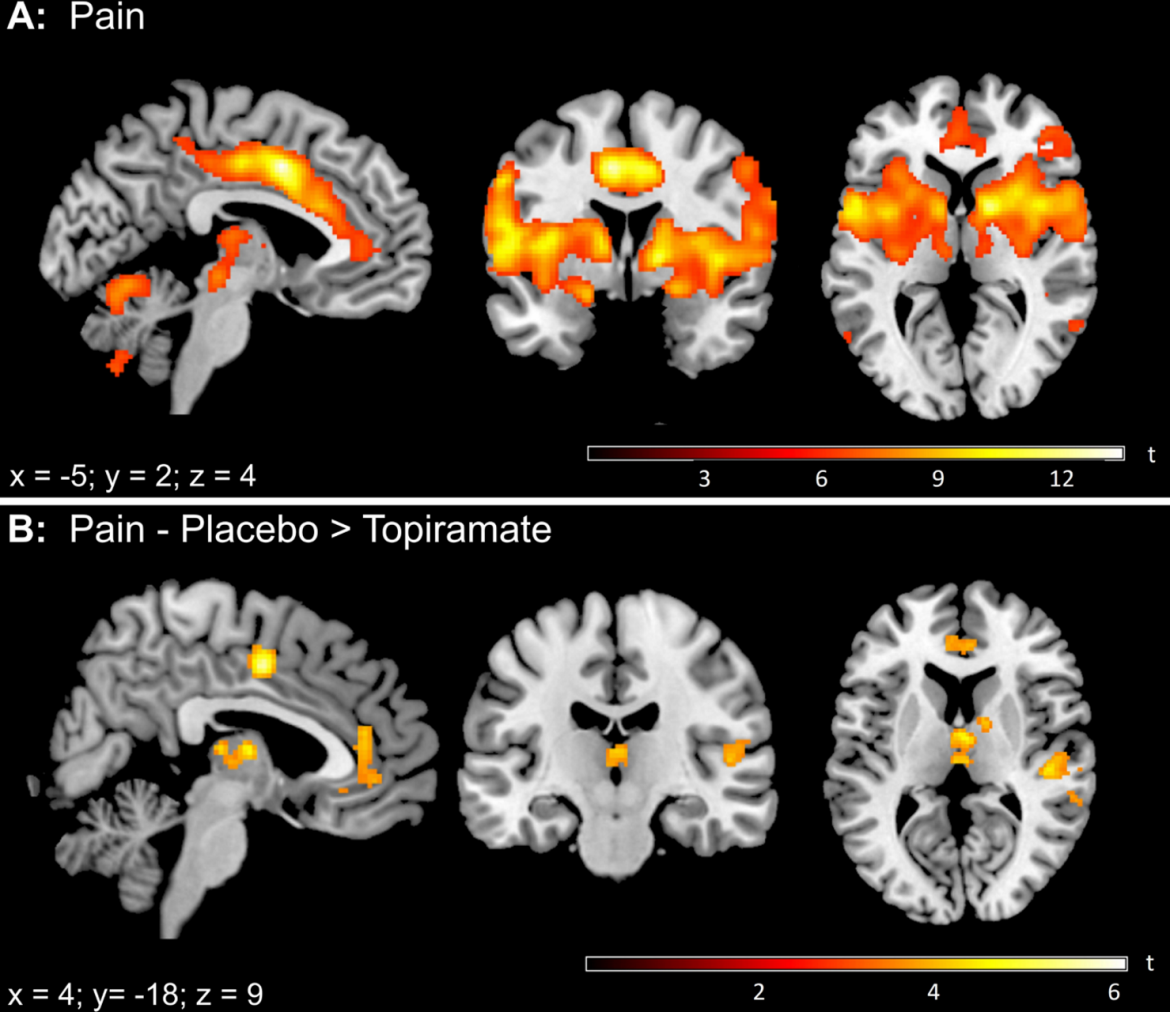
BOLD activation pattern during nociceptive input (ammonia > air). (A) Thresholded SPM{t} map (*p*_(FWE)_ < 0.05) for the BOLD contrast ammonia > air across both sessions. (B) Thresholded SPM{t} map (*p*_(FWE)_ < 0.05) for the BOLD contrast placebo > topiramate. Increased BOLD signal intensity was detected in the thalamus, anterior cingulate cortex, midcingulate cortex and the secondary somatosensory cortex during placebo compared to topiramate.

During noxious ammonia stimulation throughout both sessions, we observed significantly increased BOLD responses (voxel level FWE-corrected) in several areas typically involved in trigeminal nociceptive processing. These areas included the bilateral thalamus, insular cortex, amygdala, midcingulate cortex (MCC) and anterior cingulate cortex (ACC), cerebellum and brainstem areas (Fig 1A).

#### Differences in pain processing between topiramate and placebo

When comparing both sessions, we found a significant increase of activation of the thalamus after placebo administration, compared to topiramate (x = 4, y = -8, z = 8, t22 = 4.72, p < 0.05 SVC). We additionally found an increased activity within the bilateral ACC, rostral anterior cingulate cortex (rACC), MCC and the secondary somatosensory cortices (Fig 1B). Full details on cluster size, coordinates and statistical values are given in Table 1. No cluster reached significance for the opposite effect (Pain x topiramate > placebo).

**Table 1:** Changes in pain processing.

			MNI coordinates			
Anatomical Region	L/R	cluster size	x	y	z	T score
**(A) placebo > topiramate (Small volume correction of the thalamus)**						
Thalamus	R	158	4	-8	8	4.72
**(B) placebo > topiramate (whole brain)**						
Secondary somatosensory cortex	L	355	-36	-32	26	6.15
Anterior cingulate cortex	R	246	8	-2	46	5.79
Secondary somatosensory cortex / Insula	R	203	48	-24	6	4.95
Lateral occipital gyrus	R	227	28	-68	22	4.94
Thalamus	R	217	4	-8	8	4.72
Anterior cingulate cortex	L	454	-2	42	-4	4.56

Peak coordinates for the contrast placebo > topiramate during nociceptive stimulation: (A) Small volume correction of the thalamus at a threshold of p_(FWE)_ < 0.05 and (B) whole brain contrast placebo > topiramate at a threshold of p_(FWE)_ < 0.05 (cluster level).

#### Psychophysiological interaction

**Fig 2:**
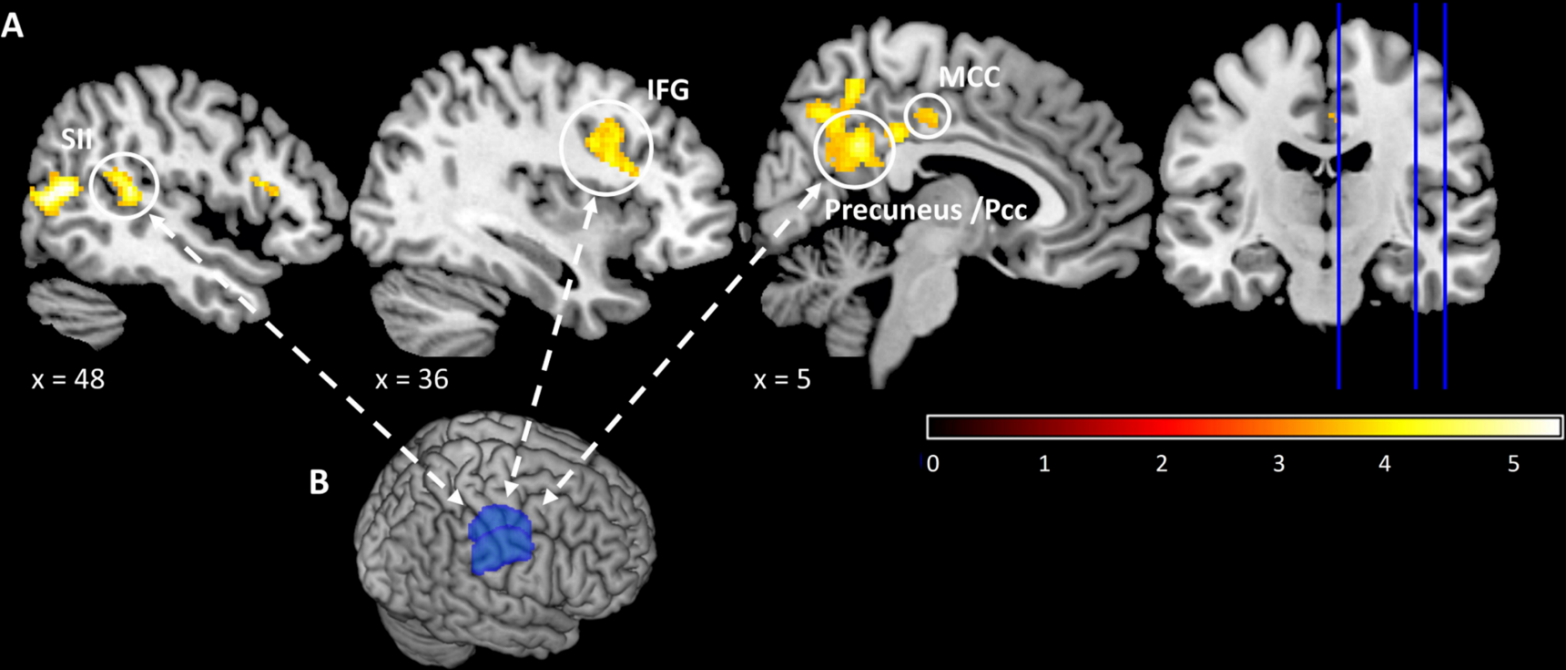
Increased thalamic connectivity under topiramate during trigeminal pain. (A) Increased coupling of the thalamus with the precuneus, postcingulate cortex (PCC), midcingulate cortex (MCC), inferior frontal gyrus (IFG) and the secondary somatosensory cortex (SII). (B) The seed region for the psychophysiological interaction analysis (PPI) was a thalamus mask of the right and left thalamus cortex of the Harvard-Oxford cortical/subcortical atlas.

To further assess the underlying network affected by topiramate, we used the thalamus as a seed region for a psychophysiological interaction (PPI) analysis [18]. Under topiramate, the thalamus showed enhanced functional coupling with the precuneus, PCC, MCC, and the secondary somatosensory cortex (p_(FWE)_ < 0.05, cluster level), when compared to placebo (Fig 2). The opposite contrast (interaction x placebo > topiramate) did not reveal any significant activation differences. All PPI results are summarized in Table 2.

**Table 2:** Thalamic connectivity during pain.

			MNI coordinates			
Anatomical Region	L/R	cluster size	x	y	z	T score
Lateral occipital cortex	R	542	50	-62	12	6.69
Inferior frontal gyrus	R	298	46	16	14	5.48
Angular gyrus	L	2555	-56	-58	38	5.45
Middle temporal gyrus	L	407	-64	-36	2	4.69

Peak coordinates for the PPI contrast topiramate > placebo at a threshold of p_(FWE)_ < 0.05 (cluster level). The big clusters encompass the precuneus, postcingulate cortex (PCC), midcingulate cortex (MCC), inferior frontal gyrus (IFG) and the secondary somatosensory cortex. A mask of the right and left thalamus cortex from the Harvard-Oxford cortical/subcortical atlas was used as a seed.

## Discussion

Our main finding is that topiramate reduces the BOLD effect in several pain transmitting brain areas including the thalamus during nociceptive trigeminal input. Topiramate also increases the functional connectivity of the thalamus to other brain areas of the somatosensory system including the precuneus. This suggests that the effect of attack reduction by topiramate in frequent migraine is not simply a question of where topiramate binds in the brain, but rather of how the presence of topiramate changes network activity.

Trigeminovascular thalamic neurons serve as a relay station for incoming nociceptive signals from brainstem areas as well as multisensory information [19]. Central sensitization of central trigeminal and thalamic neurons can alter the perception of somatosensory trigeminovascular inputs and thereby contribute to the development of cutaneous allodynia [20]. The current findings thus corroborate animal studies suggesting that migraine-effective anticonvulsants aim at nociceptive trigeminothalamic and trigeminovascular pathways [21].

Given the finding of an inhibiting effect on a specific thalamo-cortical network, it could be presumed, that topiramate binds at the thalamus and thereby interrupts the connectivity to the cortex. But analyzing changes in functional connectivity, we did not find any connection between the seed region (thalamus) and other pain-related areas that was interrupted or inhibited by topiramate. On the contrary we found that topiramate increases the functional connectivity of the thalamus to other brain areas of the somatosensory system including the precuneus. An abnormal communication between pain-modulating areas in migraineurs has been demonstrated [22–24]. Thalamo-cortical dysrhythmia is imminent to this atypical information processing [25,26] and plays a major role in migraine pathophysiology [7]. Consequently dysfunctional thalamic connectivity presents itself as a fruitful target for preventive medication. Topiramate potentially interrupts, changes or corrects patterns of dysfunctional connectivity present in the migraine brain. This altered functional coupling might be consolidated over time and eventually facilitate the preventive effect of topiramate in migraine. The psychophysiological interaction analysis does not allow for directional interpretation, it rather shows that activation in those areas oscillate in synchrony. The enhanced functional coupling between the thalamus and several brain regions involved in somatosensory processing under topiramate, might therefore constitute the migraine preventive effect, representing the normalization of dysrhythmic functional coupling. Nevertheless this speculation could only be tested with longitudinal approaches, ideally as a connection of fMRI and resting state connectivity or arterial spin labeling which would offer further insight into neuronal connectivity and help to identify network alterations caused by topiramate.

This network, or systems effect of topiramate, would also be consistent with its ability to elicit well known side effects such as cognitive impairment. Cognitive side effects of topiramate are well known and have been described in epilepsy patients [27], migraineurs [28] and healthy subjects [29]. These deficits are reported after single-dose treatment and on steady-dose and encompass reduced attention, psychomotor-speed, working memory and language impairment [29]. Some studies also assessed the effects of topiramate on fMRI [30,31]. Investigating the effect of topiramate on verbal fluency MRI, a positive correlation of topiramate dose with precuneus activity was demonstrated [32].

### Study limitations

We note that no modulatory effect of topiramate on pain perception was found. This was expected, given that topiramate has no antinociceptive properties in the clinically used dose range and it is not used as a pain killer. Another reason why we have not seen a modulatory effect on pain perception, is that the short nature of the experimental pain stimulus differs perceptively from migraine pain. Moreover, in clinical practice, we see that pain modulating effects of preventive medication are often delayed. Our study focusses on central effects of the usual dosage used to treat migraine patients, and our observation of the acute effects of topiramate may shed light on the question how topiramate exerts its effects in migraine treatment. Furthermore, as topiramate is a preventive medication, its long term effects are of central importance. Besides, the effect of topiramate was studied in the healthy system as opposed to the pathophysiological system of migraineurs. At the outset this was crucial as changes in the migraineurs brain caused by topiramate, do not enable us to draw conclusions as to what caused the change: the medication directly, or the reduction of pain caused by the medication. Thus future clinical research should focus on the central effects of topiramate in the pathological system of migraine patients.

## Conclusion

Our results corroborate evidence from animal studies suggesting that topiramate modulates nociceptive trigeminal transmission [12] with data from healthy human subjects. Topiramate attenuated the pain-related activity of parts of the thalamo-cortical network and enhanced thalamic connectivity to the precuneus, PCC and SII. These results suggest that topiramate targets a system, rather than a single structure. Moreover they could represent changes in (dys)function that consolidate over time constituting to the preventive effect in migraine. As the current study investigated healthy participants, future investigations in a patient’s cohort are needed. Pinpointing the mode and locus of action of effective medication will enhance our understanding of migraine pathophysiology.

## References

[1] Steiner TJ, Stovner LJ, Birbeck GL. Migraine: the seventh disabler. Cephalalgia Int J Headache. 2013;33: 289–290. doi: 10.1177/0333102412473843 23307815 10.1177/0333102412473843

[2] Vos T, Allen C, Arora M, Barber RM, Bhutta ZA, Brown A, et al Global, regional, and national incidence, prevalence, and years lived with disability for 310 diseases and injuries, 1990–2015: a systematic analysis for the Global Burden of Disease Study 2015. The Lancet. 2016;388: 1545–1602. doi: 10.1016/S0140-6736(16)31678-6 27733282 10.1016/S0140-6736(16)31678-6 PMC5055577

[3] D’Amico D, Usai S, Grazzi L, Rigamonti A, Solari A, Leone M, et al Quality of life and disability in primary chronic daily headaches. Neurol Sci Off J Ital Neurol Soc Ital Soc Clin Neurophysiol. 2003;24 Suppl 2: S97–100. doi: 10.1007/s100720300052 12811603 10.1007/s100720300052

[4] Brandes JL, Saper JR, Diamond M, Couch JR, Lewis DW, Schmitt J, et al Topiramate for migraine prevention: a randomized controlled trial. Jama. 2004;291: 965–73. doi: 10.1001/jama.291.8.965 14982912 10.1001/jama.291.8.965

[5] Edwards KR, Potter DL, Wu S-C, Kamin M, Hulihan J. Topiramate in the preventive treatment of episodic migraine: a combined analysis from pilot, double-blind, placebo-controlled trials. CNS Spectr. 2003;8: 428–432. 12858132 10.1017/s1092852900018733

[6] Silberstein SD, Lipton RB, Dodick DW, Freitag FG, Ramadan N, Mathew N, et al Efficacy and safety of topiramate for the treatment of chronic migraine: a randomized, double-blind, placebo-controlled trial. Headache. 2007;47: 170–180. doi: 10.1111/j.1526-4610.2006.00684.x 17300356 10.1111/j.1526-4610.2006.00684.x

[7] Goadsby PJ, Holland PR, Martins-Oliveira M, Hoffmann J, Schankin C, Akerman S. Pathophysiology of Migraine: A Disorder of Sensory Processing. Physiol Rev. 2017;97: 553–622. doi: 10.1152/physrev.00034.2015 28179394 10.1152/physrev.00034.2015 PMC5539409

[8] Shank RP, Gardocki JF, Streeter AJ, Maryanoff BE. An Overview of the Preclinical Aspects of Topiramate: Pharmacology, Pharmacokinetics, and Mechanism of Action. Epilepsia. 2000;41: 3–9. doi: 10.1111/j.1528-1157.2000.tb02163.x 10768292

[9] Schneiderman JH. Topiramate: pharmacokinetics and pharmacodynamics. Can J Neurol Sci J Can Sci Neurol. 1998;25: 3–5. 10.1017/s031716710003482x 9706732

[10] Storer RJ, Goadsby PJ. Topiramate is likely to act outside of the trigeminocervical complex. Cephalalgia Int J Headache. 2013;33: 291–300. doi: 10.1177/0333102412472069 23314783 10.1177/0333102412472069

[11] Andreou AP, Shields KG, Goadsby PJ. GABA and valproate modulate trigeminovascular nociceptive transmission in the thalamus. Neurobiol Dis. 2010;37: 314–323. doi: 10.1016/j.nbd.2009.10.007 19837163 10.1016/j.nbd.2009.10.007

[12] Andreou AP, Goadsby PJ. Topiramate in the treatment of migraine: A kainate (glutamate) receptor antagonist within the trigeminothalamic pathway. Cephalalgia. 2011;31: 1343–1358. doi: 10.1177/0333102411418259 21893557 10.1177/0333102411418259

[13] Kröger IL, May A. Triptan-induced disruption of trigemino-cortical connectivity. Neurology. 2015;84: 2124–2131. doi: 10.1212/WNL.0000000000001610 25948722 10.1212/WNL.0000000000001610

[14] Stankewitz A, Voit H, Bingel U, Peschke C, May A. A new trigemino-nociceptive stimulation model for event-related fMRI. Cephalalgia. 2010;30: 475–485. doi: 10.1111/j.1468-2982.2009.01968.x 19673914 10.1111/j.1468-2982.2009.01968.x

[15] Koban L, Brass M, Lynn MT, Pourtois G. Placebo Analgesia Affects Brain Correlates of Error Processing. El-Deredy W, editor. PLoS ONE. 2012;7: e49784 doi: 10.1371/journal.pone.0049784 23185436 10.1371/journal.pone.0049784 PMC3504079

[16] Sun J, Yang C, Zhao H, Zheng P, Wilkinson J, Ng B, et al Randomised clinical trial: the clinical efficacy and safety of an alginate-antacid (Gaviscon Double Action) versus placebo, for decreasing upper gastrointestinal symptoms in symptomatic gastroesophageal reflux disease (GERD) in China. Aliment Pharmacol Ther. 2015;42: 845–854. doi: 10.1111/apt.13334 26228097 10.1111/apt.13334 PMC5042071

[17] Contin M, Riva R, Albani F, Baruzzi A. Simple and rapid liquid chromatographic-turbo ion spray mass spectrometric determination of topiramate in human plasma. J Chromatogr B Biomed Sci App. 2001;761: 133–137. 10.1016/s0378-4347(01)00302-4 11585128

[18] Friston KJ, Buechel C, Fink GR, Morris J, Rolls E, Dolan RJ. Psychophysiological and Modulatory Interactions in Neuroimaging. NeuroImage. 1997;6: 218–229. doi: 10.1006/nimg.1997.0291 9344826 10.1006/nimg.1997.0291

[19] Noseda R, Jakubowski M, Kainz V, Borsook D, Burstein R. Cortical projections of functionally identified thalamic trigeminovascular neurons: implications for migraine headache and its associated symptoms. J Neurosci Off J Soc Neurosci. 2011;31: 14204–14217. doi: 10.1523/JNEUROSCI.3285-11.2011 21976505 10.1523/JNEUROSCI.3285-11.2011 PMC3501387

[20] Burstein R, Jakubowski M, Garcia-Nicas E, Kainz V, Bajwa Z, Hargreaves R, et al Thalamic sensitization transforms localized pain into widespread allodynia. Ann Neurol. 2010;68: 81–91. doi: 10.1002/ana.21994 20582997 10.1002/ana.21994 PMC2930514

[21] Hoffmann J, Akerman S, Goadsby PJ. Efficacy and mechanism of anticonvulsant drugs in migraine. Expert Rev Clin Pharmacol. 2014;7: 191–201. doi: 10.1586/17512433.2014.885835 24494792 10.1586/17512433.2014.885835

[22] Maleki N, Becerra L, Brawn J, Bigal M, Burstein R, Borsook D. Concurrent functional and structural cortical alterations in migraine. Cephalalgia. 2012;32: 607–620. doi: 10.1177/0333102412445622 22623760 10.1177/0333102412445622 PMC3846436

[23] Schulte LH, May A. The migraine generator revisited: continuous scanning of the migraine cycle over 30 days and three spontaneous attacks. Brain J Neurol. 2016;139: 1987–1993. doi: 10.1093/brain/aww097 27190019 10.1093/brain/aww097

[24] Yuan K, Zhao L, Cheng P, Yu D, Zhao L, Dong T, et al Altered Structure and Resting-State Functional Connectivity of the Basal Ganglia in Migraine Patients Without Aura. J Pain. 2013;14: 836–844. doi: 10.1016/j.jpain.2013.02.010 23669074 10.1016/j.jpain.2013.02.010

[25] Coppola G. Somatosensory evoked high-frequency oscillations reflecting thalamo-cortical activity are decreased in migraine patients between attacks. Brain. 2005;128: 98–103. doi: 10.1093/brain/awh334 15563513 10.1093/brain/awh334

[26] Coppola G, Iacovelli E, Bracaglia M, Serrao M, Di Lorenzo C, Pierelli F. Electrophysiological correlates of episodic migraine chronification: evidence for thalamic involvement. J Headache Pain. 2013;14: 76 doi: 10.1186/1129-2377-14-76 24016158 10.1186/1129-2377-14-76 PMC3844625

[27] Blum D, Meador K, Biton V, Fakhoury T, Shneker B, Chung S, et al Cognitive effects of lamotrigine compared with topiramate in patients with epilepsy. Neurology. 2006;67: 400–406. doi: 10.1212/01.wnl.0000232737.72555.06 16894098 10.1212/01.wnl.0000232737.72555.06

[28] Kececi H, Atakay S. Effects of topiramate on neurophysiological and neuropsychological tests in migraine patients. J Clin Neurosci Off J Neurosurg Soc Australas. 2009;16: 1588–1591. doi: 10.1016/j.jocn.2009.03.025 19793661 10.1016/j.jocn.2009.03.025

[29] Meador KJ, Loring DW, Vahle VJ, Ray PG, Werz MA, Fessler AJ, et al Cognitive and behavioral effects of lamotrigine and topiramate in healthy volunteers. Neurology. 2005;64: 2108–2114. doi: 10.1212/01.WNL.0000165994.46777.BE 15985582 10.1212/01.WNL.0000165994.46777.BE

[30] Szaflarski JP, Allendorfer JB. Topiramate and its effect on fMRI of language in patients with right or left temporal lobe epilepsy. Epilepsy Behav EB. 2012;24: 74–80. doi: 10.1016/j.yebeh.2012.02.022 22481042 10.1016/j.yebeh.2012.02.022 PMC3564045

[31] Wandschneider B, Burdett J, Townsend L, Hill A, Thompson PJ, Duncan JS, et al Effect of topiramate and zonisamide on fMRI cognitive networks. Neurology. 2017;88: 1165–1171. doi: 10.1212/WNL.0000000000003736 28213372 10.1212/WNL.0000000000003736 PMC5373787

[32] Yasuda CL, Centeno M, Vollmar C, Stretton J, Symms M, Cendes F, et al The effect of topiramate on cognitive fMRI. Epilepsy Res. 2013;105: 250–255. doi: 10.1016/j.eplepsyres.2012.12.007 23333471 10.1016/j.eplepsyres.2012.12.007 PMC3725414

